# An Automated Versatile Diagnostic Workflow for Infectious Disease Detection in Low-Resource Settings

**DOI:** 10.3390/mi15060708

**Published:** 2024-05-28

**Authors:** Miren Urrutia Iturritza, Phuthumani Mlotshwa, Jesper Gantelius, Tobias Alfvén, Edmund Loh, Jens Karlsson, Chris Hadjineophytou, Krzysztof Langer, Konstantinos Mitsakakis, Aman Russom, Håkan N. Jönsson, Giulia Gaudenzi

**Affiliations:** 1 Department of Global Public Health, Karolinska Institutet, 17177 Stockholm, Sweden; miren.urrutia@scilifelab.se (M.U.I.); phuthumani.mlotshwa@ki.se (P.M.); jesper.gantelius@gmail.com (J.G.); tobias.alfven@ki.se (T.A.); 2 Science for Life Laboratory, Division of Nanobiotechnology, Department of Protein Science, KTH Royal Institute of Technology, 17165 Stockholm, Sweden; krzysztof.langer@ucsf.edu (K.L.); aman.russom@scilifelab.se (A.R.); hakan.jonsson@scilifelab.se (H.N.J.); 3 Department of Microbiology, Tumor and Cell Biology, Karolinska Institutet, 17165 Stockholm, Sweden; edmund.loh@ki.se (E.L.); jens.karlsson@ki.se (J.K.); chris.hadjineophytou@ki.se (C.H.); 4 Hahn-Schickard, Georges-Koehler-Allee 103, 79110 Freiburg, Germany; konstantinos.mitsakakis@hahn-schickard.de; 5 Laboratory for MEMS Applications, IMTEK–Department of Microsystems Engineering, University of Freiburg, Georges-Koehler-Allee 103, 79108 Freiburg, Germany

**Keywords:** modular automation, open-source, recombinase polymerase amplification, microarray, signal enhancement, infectious diseases

## Abstract

Laboratory automation effectively increases the throughput in sample analysis, reduces human errors in sample processing, as well as simplifies and accelerates the overall logistics. Automating diagnostic testing workflows in peripheral laboratories and also in near-patient settings -like hospitals, clinics and epidemic control checkpoints- is advantageous for the simultaneous processing of multiple samples to provide rapid results to patients, minimize the possibility of contamination or error during sample handling or transport, and increase efficiency. However, most automation platforms are expensive and are not easily adaptable to new protocols. Here, we address the need for a versatile, easy-to-use, rapid and reliable diagnostic testing workflow by combining open-source modular automation (Opentrons) and automation-compatible molecular biology protocols, easily adaptable to a workflow for infectious diseases diagnosis by detection on paper-based diagnostics. We demonstrated the feasibility of automation of the method with a low-cost *Neisseria meningitidis* diagnostic test that utilizes magnetic beads for pathogen DNA isolation, isothermal amplification, and detection on a paper-based microarray. In summary, we integrated open-source modular automation with adaptable molecular biology protocols, which was also faster and cheaper to perform in an automated than in a manual way. This enables a versatile diagnostic workflow for infectious diseases and we demonstrated this through a low-cost *N. meningitidis* test on paper-based microarrays.

## 1. Introduction

The COVID-19 pandemic has underscored the importance of well-equipped and efficient diagnostic laboratories [1]. Operating under constant pressure to improve quality and efficiency, diagnostic laboratories face challenges such as personnel shortages and the need for constant workflow evaluation [2]. Adopting novel technologies becomes crucial for enhancing the quality and reducing turnaround times of tests [3]. Particularly in resource-limited settings, advancements in diagnostic tests, integration of robotics and automation, and making use of frugal innovations are essential for accuracy, efficiency, and accessibility.

Laboratory automation plays a key role in enhancing the quality and efficiency of testing [4,5,6]. By automating repetitive tasks and enabling the simultaneous processing of multiple samples, automation makes laboratory work more efficient [7]. It reduces dependency on highly-trained personnel and helps to minimize human errors. Integrating automation platforms is particularly beneficial for diagnostics laboratories, where infectious samples are routinely handled, and accurate, rapid diagnostic results are critical [8,9,10]. Furthermore, laboratory automation increases operational capacity in clinical workflows, improves logistics, and enables onsite testing and rapid decision-making in patient management.

Diagnostic tests are vital for the early and accurate detection of infectious pathogens, but the diagnostic standards of care are often expensive, complex and labor-intensive. The need for rapid, easy-to-use and adaptable tests that can be deployed in diverse settings has become evident, especially during outbreaks and cross-border disasters. Automated diagnostic tools can address these needs by increasing capacity, eliminating the impact of human operational errors on results, and optimizing personnel resources. They can therefore bridge the gap between point-of-care testing and central laboratories, meeting the demand for mid-throughput testing.

Various automation platforms, including liquid-handling robots (LHR), have gained prominence within laboratory settings over the years [4,11]. LHRs, exemplified by Flow Robotics robots, streamline the analysis of tests by executing laboratory protocols without supervision [12]. Automated laboratory workflows, such as ELISA assays using cartridges [13], and robotic digital microfluidics for urinalysis tests on disposable superhydrophobic cartridges [14], often lack open-source software interfaces, hindering protocol customization, interoperability and rapid adaptability, which are crucial in cases of epidemics [15]. High procurement and maintenance costs and specific labware requirements also hinder accessibility [4,16].

To address these challenges, we propose a modular workflow compatible with Opentrons OT One-S Hood, an open-source liquid-handling robot. This low-cost platform which offers an open-source application programming interface (API), facilitates accessibility, adaptability to different pathogens and settings, and widespread standardization of protocols. The feasibility of integrating several modules into an automated workflow was demonstrated using *Neisseria meningitidis*, a pathogen that is prone to causing endemic and epidemic infections in young and healthy adults worldwide [17]. Furthermore, a resources analysis was carried out with assessment of the time and cost that were required to carry out the necessary steps of the workflow in the presented automated way, compared to a manual manner, in an attempt to assess the potential of the developed workflow to be deployed to resource-limited settings, where the affordability (cost), the workflow time (e.g., in case of epidemics), but also the throughput (samples analyzed per run) are essential characteristics for a diagnostic system.

## 2. Materials and Methods

**Figure 1:**
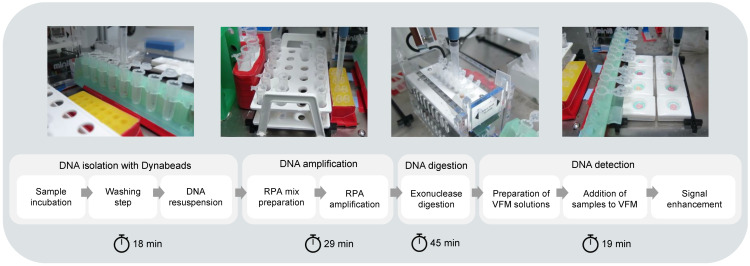
The automated workflow for *N. meningitidis* detection using the OT-One Hood robot consists of four modules: (1) the ‘DNA isolation with Dynabeads’, (2) the ‘DNA amplification’, (3) the ‘DNA digestion’, and (4) the ‘DNA detection’ module. All the steps were performed automatically by the robot, except for the opening and closing of tube lids before and after the DNA amplification, and the exonuclease digestion steps on the MiniPCR^®^ mini8 thermal cycler. The indicated times are as programmed.

The workflow was set up as depicted in Figure 1 and consisted of four modules, beginning with the DNA from the sample binding to magnetic beads, followed by a washing step to remove sample debris and then releasing the DNA from the beads in a resuspension buffer using repeated pipetting. The purified DNA and the reagents required for the amplification reaction were mixed at 37 °C, followed by digestion of the double-stranded amplicons obtained from the amplification reaction at 37 °C. The products of DNA digestion were then pipetted onto the paper-based microarrays and the signal was enhanced with a method based on gold nanoparticles.

### 2.1. DNA Isolation

DNA from *N. meningitidis* was obtained using the Wizard^®^ Genomic DNA Purification Kit (Promega, Madison, WI, USA) according to manufacturer’s instructions with the final DNA being eluted in DNAse-free water (NFW) (Invitrogen; cat. no. AM9935, now ThermoFisher, Waltham, MA, USA), and cerebrospinal fluid (CSF) at a final concentration of 1000 ng/µL. The CSF matrix was chosen as it is usually the specimen associated with *N. meningitidis* detection in clinical samples. The DNA in spiked samples was isolated using the Dynabeads DNA Universal Kit (ThermoFisher, Waltham, MA, USA, cat. no. 63006), following the manufacturer’s instructions. The isolated DNA was eluted from the Dynabeads in 40 µL of NFW (Figure 1). 

### 2.2. ctrA Gene Amplification

After isolating the *N. meningitidis* DNA, the samples were subjected to isothermal recombinase polymerase amplification (RPA). Here, the *ctrA* gene from *N. meningitidis* was amplified using primers (Eurofins Genomics, Ebersberg, Germany) previously described by Rivas et al. [18] and displayed in Table 1. The reverse primer carried a phosphate group at the 5′-end, whilst the forward primer was biotin-labelled at the 5′-end. These modifications were key for the subsequent enzymatic digestion of the amplicons to obtain single-stranded DNA and detect the amplified gene on the microarray, respectively. The RPA reaction was set up using the TwistAmp^®^ Basic Kit (TwistDx, Maidenhead, UK cat. no. TABAS03KIT) and following the manufacturer’s instructions. In brief, eight reactions were prepared by mixing the primers, rehydration buffer, template or water and magnesium acetate into a TwistAmp^®^ Basic reaction tube. Instead of vortexing, the robot was programmed to pipette the reactions without causing froth. Samples were heated to 37 °C in the MiniPCR^®^ mini8 thermal cycler (miniPCR bio^®^, Cambridge, MA, USA) for 20 min. To confirm the successful amplification of the desired DNA fragment, 1 µL of the amplified DNA solution was analyzed using the Agilent DNA 1000 Kit (cat. no. 5067-1505) and following the manufacturer’s instructions on a BioAnalyzer chip.

### 2.3. Enzymatic Digestion of Amplicons

Following the amplification of the *ctrA* fragments, the double-stranded amplicons were enzymatically digested to obtain single-stranded DNA. This was done by incubating the solutions obtained from the amplification step with 10 μL of Lambda Exonuclease 5U (Thermo Fischer; cat no. EN0561), an enzyme that selectively digests DNA strands tagged with a phosphate group, and leaves the biotinylated DNA strands intact. The digestion took place at 37 °C for 30 min and was followed by a 10-min inactivation step at 80 °C. The same MiniPCR^®^ mini8 thermal cycler described was used to carry out this step. This resulted in obtaining a 50 µL solution containing single-stranded *ctrA* fragments.

### 2.4. Detection of Single-Stranded ctrA Amplicons

**Figure 2:**
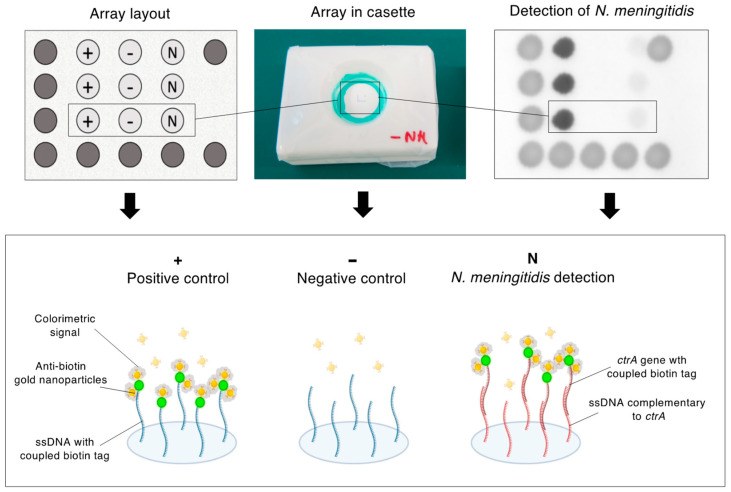
Layout of the *N. meningitidis* Vertical Flow Microarray (VFM). Synthetic probes with 5′-end biotin-TEG tag modifications are printed at the positive control spots. The negative control spots contain unmodified synthetic probes. Capture probes complementary to the *ctrA* gene from *N. meningitidis* are printed on the detection spots. The *ctrA* amplicons generated from the RPA reaction contain a 5′-end biotin-TEG tag. Anti-biotin gold nanoparticles (ab-AuNPs) bind the biotin tags and produce a colorimetric signal. A frame of ink spots is printed on the array to ensure the user handles the VFM in the correct orientation.

A vertical flow microarray (VFM) validated previously by Rivas et al. [18], was designed for the detection of *ctrA* single-stranded fragments. The microarray was printed on a nitrocellulose membrane (Protran Ba79 0.1 µm, Whatman, Maidstone, UK) using a Nanoplotter 2.1 (GeSiM, Radeberg, Germany). In short, nitrocellulose rectangles (75 mm × 25 mm) were cut and attached to standard glass microscope slides using double-sided tape. The glass slides were placed inside the printer along with a humidifier that kept the humidity constant at 40% within the printer’s hood. Maintaining high humidity within the hood helps minimize the evaporation of droplets during deposition on the membrane. Four different solutions were printed on the membranes to constitute the microarrays (Figure 2). First, an ink-containing solution was printed as a visible frame. The ink solution was prepared as described by Gökçe et al. [19] and consisted of 1 mg/mL brilliant black BN and 10% PEG-3000, diluted in Milli-Q water. 

Three columns were printed in the frame, each containing three rows of array spots. The first column contained three positive control spots with biotinylated synthetic DNA oligonucleotides. The second column contained three negative control spots with synthetic DNA oligonucleotides. The third column contained the capture probes for the amplified *ctrA* gene of *N. meningitidis*. The sequences of the synthetic DNA oligonucleotides and the capture probes are presented in Table 2. In total, 3 nL of DNA-containing solution were printed at each spot on the microarray, which corresponds to 10 droplets of solution per spot. Each spot printed on the array had a diameter of 200 μm. After printing, the membranes were left inside the printer to dry overnight at room temperature (RT). 

After drying, the microarrays were placed in a holder filled with sponges for testing the solutions containing the amplified DNA. A PAP hydrophobic pen (Sigma Aldrich, St. Louis, MO, USA; cat. no. Z672548) was used to draw a circle around the microarray to ensure the liquid pipetted onto it moved horizontally to the bottom layers. First, 100 µL of 3% bovine serum albumin (BSA) blocking buffer (Sigma-Aldrich; cat.no. A7905-50G) in Tris Buffered Saline (TBS) was added to the microarray. Then, the amplified DNA (or the negative control solution) was diluted into 150 µL of TBS and pipetted onto the membrane. The array was then subjected to a washing step, in which 100 µL of 0.05% Tween-20 in TBS were pipetted onto the array. After washing, 100 μL of a solution containing monoclonal anti-biotin coated gold nanoparticles (AuNPs) (BBI Solutions) (AuNPs 1:4 in TBS) were added to the microarray. A final washing step was carried out to remove unbound AuNPs from the membranes.

The signal enhancement procedure followed. The signal enhancement solution was a mixture of NFW, 10 mM MES (pH 6), 30% 9.8 M H_2_O_2_, and 50 mM HAuCl_4_ in the proportion 7:1:1:1. This solution was freshly prepared just before being applied onto the arrays. 500 μL of the prepared solution was pipetted onto each of the microarrays. The nitrocellulose membranes were rinsed briefly in water. After washing, the arrays were air dried at RT and were scanned using the flatbed scanner CanoScan 9000F Mark II (Canon, Tokyo, Japan) in 16-bit grayscale.

### 2.5. Analysis of the Images

The images from scanning the microarrays were analyzed and quantified using the Fiji Software from NIH (version 2.0.0-rc-69/1.52p). The signal-to-noise ratio was obtained by separately calculating the mean signal intensities from the positive control, negative control, and template spots.

### 2.6. Automation of the Workflow

The LHR used for automating the operation of the VFM was the Opentrons OT-One-S Hood (Opentrons). The LHR was connected to a laptop on which the Opentrons software was installed. The latter was used to initialize the robot, although it was not required for its operation. Instead, a program was developed by the Nanobiotechnology division at SciLifeLab to facilitate the creation of scripts for experimental protocols [20]. 

### 2.7. Platform Setup

**Figure 3:**
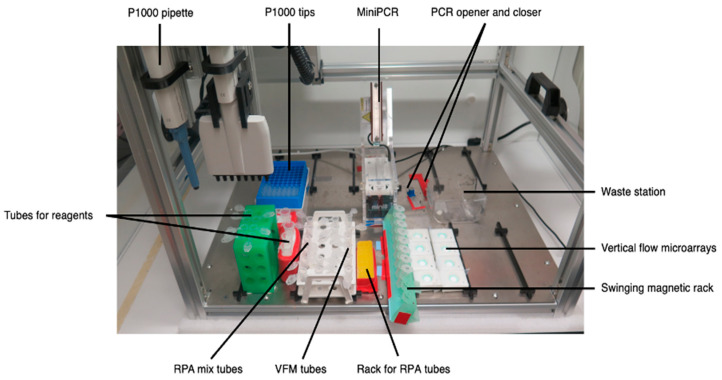
Photograph showing the set-up for the diagnostic workflow on the OT-One Hood robot. A MiniPCR^®^ mini8 was used as a thermocycler for the amplification and enzymatic digestion steps.

Before initiating sample analysis, the materials and reagents needed were placed on the LHR platform (Figure 3). This included a newly opened 1 mL tip rack, the miniPCR^®^ mini8 thermal cycler, the thermal cycler’s opening and closing custom-made tools, a container for waste and used pipette tips, a rack with seven 1.5 mL Eppendorf tubes and six 5 mL Eppendorf tubes containing some of the needed reagents, a rack with six larger tubes, a rack with 16 × 1.5 mL Eppendorf tubes for preparing the samples for the RPA and the VFM procedures, a rack with eight tubes containing the lyophilized enzymes needed for the RPA reaction, a swinging rack with eight tubes to perform the DNA isolation step with the magnetic beads and, finally, eight microarrays placed in the holders as previously described. In addition, eight 0.5 mL tubes were placed inside the thermal cycler. The following reagents were placed in the 1.5 mL Eppendorf tubes: the primer mix, the resuspension buffer for the RPA reaction, the MgOAc solution for starting the amplification reaction, the Lambda Exonuclease solution, the blocking buffer, the gold-nanoparticle solution for the VFM procedures, and a 1X TBS solution. The reagents that were prepared in the 5 mL tubes were the 1X washing buffer, resuspension buffer for the DNA isolation protocol, nuclease-free H_2_O, the washing buffer for the VFM procedure, the signal-enhancing solution for the VFM, and PBS.

## 3. Results

The diagnostic workflow for *N. meningitidis* that we have developed is composed of four main modules. The first module consists of the isolation of DNA from a sample using magnetic beads. In the second module, an RPA reaction has been optimized for the specific amplification of the *ctrA* gene, which is unique to *N. meningitidis*. In the third module, amplicons derived from this process are digested into single-stranded DNA in the presence of exonucleases. Lastly, in the fourth module, a VFM test is used to detect the presence of single-stranded *ctrA* fragments in the sample. For this, a colorimetric signal enhancement assay using gold nanoparticles is employed. These four modules have been optimized and adapted to be carried out entirely through automated liquid handling.

### 3.1. Results from the RPA Module

**Figure 4:**
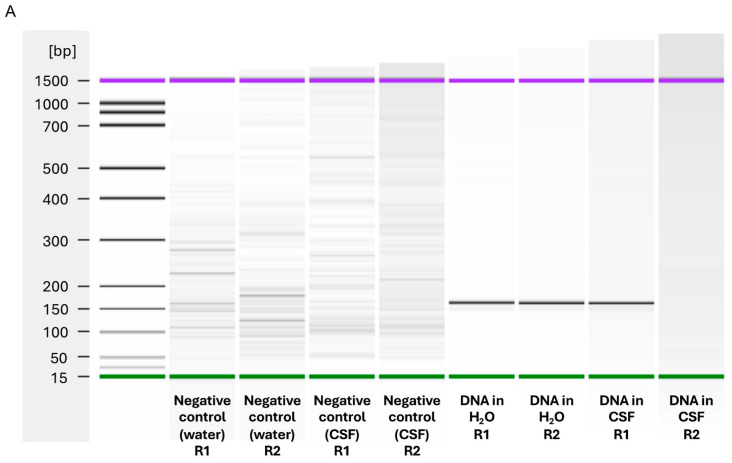

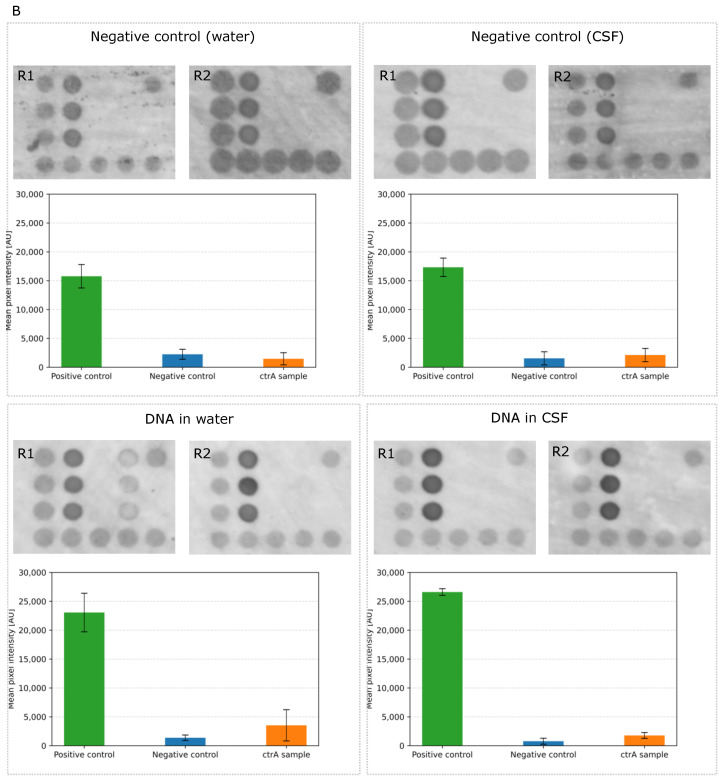
(**A**) Gel electrophoresis results from the Agilent Bioanalyzer for an RPA reaction, showing amplified products from the spiked samples. Both spiked water replicates (R1 and R2) showed clear amplification while only one spiked CSF sample replicate (R1) showed amplification. (**B**) Scans of the VFM after performing the signal enhancement procedure, with the corresponding signal intensity measurement graphs obtained from the analysis of the membranes. Color changes were not observed on the *ctrA* microarray spots for CSF spiked samples, while the color change was visible for spiked water samples. The error bars are derived from averaging the three R1 and three R2 spots. R2 samples were replicates of R1 samples.

The samples obtained from the RPA reaction were analyzed by electrophoresis using a BioAnalyzer instrument. This was done in parallel with the automated workflow to verify whether any nucleic acid amplification occurred during the reaction. The BioAnalyzer gels obtained show the amplification of the 146 base pair fragment that corresponds to the *ctrA* gene (Figure 4A). This amplification was observed on samples spiked with *N. meningitidis* DNA as expected and not in the negative controls. The nuclease-free water spiked with *N. meningitidis* showed clear amplification on the gel in both replicates R1 and R2, while the second replicate R2 of the spiked CSF did not show any amplification. 

### 3.2. Results from the VFM Module

The paper arrays on which spiked samples in water were added showed satisfactory results, as both the positive control and the sample spots showed clear hybridization signals after being subjected to the signal enhancement procedure. The negative control spots remained pale (Figure 4B). The results for the spiked samples in CSF were less successful: even though the positive-control spots were colored as expected, the sample spots did not show signs of *ctrA* hybridization. This was expected for the second replicate (R2), as no amplification had been detected on the Bioanalyzer gel. The VFMs on which the negative control samples were added worked as expected.

### 3.3. Functionality

#### 3.3.1. Time

**Figure 5:**
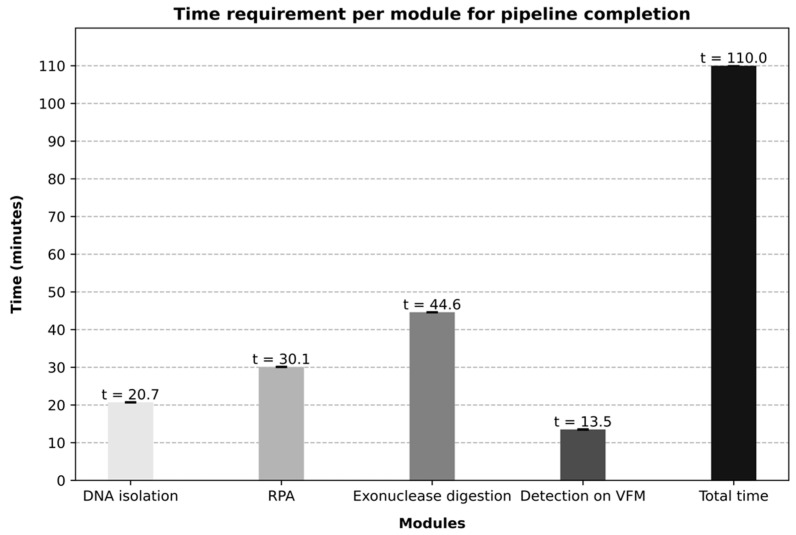
Average estimation of the time required for the liquid-handling robot to analyze eight samples in parallel. The time requirement of each of the modules was quantified over five independent experiments.

The time required for the liquid-handling robot to carry out the entire workflow for eight samples was 110 min (Figure 5). This estimation comprises the time required from the moment the samples are added to the Dynabeads lysing solution until the final washing step carried out by the robot after the signal enhancement procedure. The preparation of the spiked samples is not accounted for in this estimation, nor is the final imaging of the arrays. In comparison, the time required for the workflow to be performed for eight samples on a laboratory bench by an experienced operator was 134 min on average.

#### 3.3.2. Cost

The cost per run for the eight samples (Table 3) was approximately USD126, assuming they were run simultaneously. Therefore, the cost to run each sample would be around USD16. The costs for most lab reagents used such as water and nitrocellulose membrane paper were considered negligible. The cost of the custom-made racks, the thermal cycler custom-made opening and closing tools, and the waste container were not considered either, as they were made from plastic labware that would have otherwise been discarded as waste. The table does not include the cost of the BioAnalyzer instrument, the correspondent chips, the NanoPlotter printer, and the computer used to instruct the liquid-handling robot. The information is correct as of January 2023.

## 4. Discussion

This work aimed to demonstrate the automation of a complete diagnostic workflow, from DNA isolation to detection. The system uses a multitude of converging and enabling technologies such as microparticles, microarrays, spotting and hybridization chemistries and technologies, hybrid system integration between microcomponents and bio/chemical materials, handling of µL-volumes, and lastly, touches upon manufacturing perspectives. 

Some limitations of the study were the fact that (1) we used spiked instead of real samples and (2) we used high concentrations of DNA and no dilution series. This is because the analytics per se (reproducibility, limit of detection, etc.) of the VFM, have been extensively described in previous work and were not the main focus of this work. Instead, the focus was to demonstrate the feasibility of a structural and functional integration, and compatibility of several different modules (including the VFM, as one of them) interfaced by a robotic liquid handling arm towards an automated workflow. An equally important focus was the quantitative assessment of the non-analytical consequences of using such a system at routine settings (e.g., time and cost) thereby assessing its overall (not only analytical) potential for implementation in resource-limited settings.

Within this context, we successfully prototyped, implemented and tested it for the detection of *N. meningitidis*. The automated workflow handled eight samples in parallel. The time required for these eight samples to be processed was less than what an experienced laboratory researcher would employ to conduct the same protocol. The estimated test cost for each of the eight samples (Table 3: USD126/8 = ~USD16) is also way less than estimated for a meningitis PCR test, which costs approximately USD94 [21], although this cost might vary from region to region.

The automated RPA module was demonstrated successfully as amplification was observed from the spiked samples. The observed difference in amplification results between the water and the CSF-spiked samples might be due to the 1000 µL pipette which has limitations in pipetting small volumes. Indicatively, the volume of the template that had to be pipetted was 5 µL, which may account for some precision issues of the 1000 µL pipette, and consequently the false negative outcome (non-amplification). Such issues can be solved by having a more sophisticated pipetting configuration, with at least two types of interchangeable tips (e.g., of 1000 µL and 100 µL volume). Effective nucleic acid extraction to increase nucleic acid purity before RPA is crucial to detection accuracy and decreasing false negative rates [22]. Achieving optimal extraction from complex sample matrices such as CSF might then require cumbersome peripheral equipment and sample processing. The solution to this challenge might be through microfluidics and its ability to separate sample components thus decreasing sample complexity before amplification [23]. Other studies that have employed RPA in detecting nucleic acid targets in complex samples have used membrane separation as well as applying electric fields for the isolation and concentration of the nucleic acids [19]. 

The decreased time spent by the automated workflow compared to the manual workflow shows the initial saving in time that can be achieved by implementing such an automated system. This would allow the laboratory personnel to carry out other activities, while the automated analysis runs. With iterative optimization of the system, less time could be achieved for this workflow. Of all the stages, nucleic acid extraction would constitute the most crucial one that may take the most time. To achieve optimal results with less complexity and cost, there is a need to identify and optimize nucleic acid extraction protocols that achieve a balance between the necessary extraction efficiency and compatibility with the automation workflow.

The development of stable, scalable and cost-effective diagnostic methods is crucial for effective disease management, especially in low-resource settings. Developing reproducible, automated testing workflows can help ensure consistent and accurate results, especially in emergencies, such as during epidemics and pandemics, where repetitive tasks become prone to human errors when handling mid- to high-throughput samples in a manual way. The automation reduces exposure to contagious infections, such as *N. meningitidis* infection, while it enables laboratory workers to focus on quality control and process improvement. Automation has further been cited to reduce sample contamination as compared to manual operations [24]. 

Several automation platforms are currently in the market and have been elucidated, with Tecan and Hamilton leading in state-of-the-art; Analytik Jena and Agilent in multi-device platforms; and Gilson, Bio Molecular Systems and Opentrons in modular platforms [(24)]. However, reliance on closed commercial platforms can limit scalability and flexibility. Research that explores the use of open-source platforms therefore offers greater flexibility and the ability to adopt external solutions to the platform. Modular open-source platforms have the potential to reduce the high cost of automation and make it accessible to laboratories with lower budgets. These platforms allow the use of standard commercially available pipette tips and reaction tubes, as opposed to specific labware, and can be combined with modules to create a diagnostic workflow that fits the needs of a particular protocol or laboratory. Because the robot is released as open-source hardware, it can be customized and repaired independently of the supplier. In cases where personnel for such tasks are not present, remote repair solutions could be explored. Furthermore, a system like the one described is largely adaptable when different biochemical protocols need to be integrated (e.g., for respiratory tract infections, urinary tract infections, sepsis, etc.) which is never the case with microfluidic platforms where for each clinical scenario there has to be a dedicated, new development, which is time-and resource-consuming, and especially unsuitable for responding to urgent needs such as in epidemic cases.

The concept of frugal innovation is also becoming increasingly important, as it emphasizes the need to create low-cost, high-impact solutions that can be adapted to different contexts, particularly in low-resource settings. Furthermore, the fact that our solution is microfluidic-free and relies only on standard, commercially available consumables like pipette tips and tubes, for which there are already established logistics pipelines for supply in resource-limited settings, contributes to the reduced development cost and the acceleration towards commercialization and implementation because there is no need for the (costly and time-consuming) design transfer steps that are typically required for a microfluidic solution to reach product development level. Furthermore, the complexity of heterogeneous integration that inherently lies within microfluidic-based diagnostics is avoided. Lastly, such microfluidic-free solutions do not require any expensive, sophisticated manufacturing lines, which means that they are more suitable for technology transfer to and implementation by low- and middle-income countries.

This study was aiming at a proof-of-concept of automating a combination of modules using an open-source robot. Diagnostic accuracy was not the focus of this study, as validated previously [18], so reliability and reproducibility of the workflow were not investigated. Establishing a reproducible workflow using OT One-S technology can present several challenges. One major limitation is the difficulty in handling small volumes, as traditional pipettes may not be precise enough. Limited lab space can also be an issue, as the systems used, such as thermocyclers and imaging equipment, still require human intervention and can take up much space. To overcome these limitations, there is a need for automated solutions that can accurately interpret results, track samples, and control the correct aspiration and deposition of liquids. This is particularly important in clinical settings, where accurate sample tracking is essential. 

## 5. Conclusions

In this work we showed the iterative development for the feasibility assessment of the integration of an automated workflow for rapid and low-cost potential diagnostic use. The successful operation of the individual modules was shown, and the capacity to adapt and interface the different modules in an automated workflow through a robotic arm for liquid handing was demonstrated, comprising a potential improvement to some state-of-the-art solutions that currently exist in the market. We also carried out some top-level logistics assessment in terms of costs and consumed time, in comparison to manual processing, and concluded that the use of the developed system is advantageous as it performed the analysis at ~18% less time than manually and at ~5.8× less cost per sample that a representative commercial kit. Further time and cost reduction can be achieved by integrating additional functions. The workflow and platform will undergo additional validation and optimization on other complex sample matrices to demonstrate their suitability and adaptability in various clinical scenarios, such as respiratory tract, urinary, and sexually transmitted infections.

Our approach combined open-source modular automation (Opentrons) with adaptable molecular biology protocols, enabling a versatile, rapid, and reliable diagnostic workflow for infectious diseases. Our study using a low-cost *N. meningitidis* test demonstrates the feasibility of this approach, bridging the gap between cost-effective automation and accurate pathogen detection on paper-based microarrays.

The integration of the workflow shown in this study offers several advantages and possibilities. These include the use of common reagents and consumables without the need for high-cost investments for specialized equipment, which, in addition to the no need for highly skilled personnel increased throughput, and decreased overall cost, make the developed platform a potential candidate for implementation in resource-limited settings. The open-source nature of the software creates immense room for continual improvement of the individual modules as they become commercially available. Furthermore, the potential for using mobile phones coupled with software applications for the reading of imaging results would eliminate the need for specialized equipment and enable the workflow to be implemented in multiple decentralized settings. The applications could incorporate various forms of machine learning to enable the reading of results of more specialized tests in remote settings and hard-to-reach areas. This workflow shows promise for decentralizing diagnostic tests, bringing molecular diagnostics closer to multiple points of use, especially in resource-limited settings.

## Figures and Tables

**Table 1:** Sequences of the RPA primers for the amplification of *ctrA* from *N. meningitidis*.

Primer	Sequence (5′–3′)	Modification (5′)	Length (bp)	Length of Amplicon (bp)
Forward	GTC AGG ATA AAT GGA TTG CTC AAG GTT A	Biotin-TEG	28	146
Reverse	CGC ATT CGA CAC ATA CAA TAC ATC TTT A	Phosphate tag	28	

**Table 2:** Sequence of the DNA probes printed on the *N. meningitidis* Vertical Flow Microarray (VFM).

Primer	Sequence (5′–3′)	Modification (5′)	Length (bp)
Positive control probe	TGT ATT TGT CTT CGA TGA GGC CCG	Biotin-TEG	25
Negative control probe	TGT ATT TGT CTT CGA TGA GGC CCG T	Amine-C6	25
*ctrA* capture probe	CAT TCG ACA CAT ACA ATA CAT CTT TAT TCT TCA C	-	22

**Table 3:** Estimation of the cost of processing eight samples using the workflow. All the costs for consumables are rough estimates based on consumption for the experiments in this study (as of January 2023). The total cost estimation includes the consumables, the reagent kits and some laboratory reagents. The cost of lab equipment and some reusable reagent solutions is not included for the 8 samples since it was considered negligible.

Item #	Item	Example	Units Needed per 8 Samples	Unit Price (USD)	Cost per 8 Samples (USD)
(A) Consumables					
1	Tip box	Fisherbrand Filter tips	80	0.045	3.60
2	Eppendorf tubes 5 mL	VWR Tubes	6	0.491	2.94
3	Eppendorf tubes 1.5 mL	Tubes	31	0.073	2.27
4	PCR tubes	Eppendorf Tubes	8	0.188	1.50
5	Sponges	Sponge pack	2	0.056	0.11
(B) Kits					
6	Dynabeads DNA Universal Kit	Kit	1600 µL	551.00	14.70
7	TwistAmp^®^ Basic Kit	Kit	8 enzyme reaction tubes	443	36.91
8	Lambda Exonuclease	Kit	-	79.46	50.38
9	Negative control for array	-	-	-	-
10	Positive control for array	-	-	-	-
11	Capture probes for array	-	-	-	-
12	Gold nanoparticles	Bottle	-	149.21	11.93
13	Primer pair	-	-	-	-
(C) Laboratory equipment					
14	Magnetic rack	Separation rack	1	59.00	-
15	Tube racks	Racks	5	23.75	-
(D) Laboratory reagents					
16	Nuclease free water	Bottle	-	32.40	0.45
17	Ink for array	Black BN	-	91.50	-
18	PEG	Bottle	-	62.30	0.012
19	BSA	Bottle	-	117.00	0.28
20	PBS	Bottle	-	102.80	-
21	TBS	Bottle	-	113.00	0.32
22	Tween-20	Bottle	-	16.80	0.03
23	MES	Bottle	-	66.10	0.003
24	HAuCl_4_	Bottle	-	533.00	-
25	Hydrophobic pen	Pen	-	111.00	-
(E) Equipment					
26	Opentrons OT-One-S Hood *	-	-	4000.00	0.4
27	MiniPCR^®^ mini8 thermal cycler *	Cycler	-	695.00	0.07
Total cost					~USD126

* Based on the assumption of a run time of 5 years at 300 days per year and 12 h per day in operation for the robot and the mini8 thermal cycler.

## Data Availability

The original contributions presented in the study are included in the article, further inquiries can be directed to the corresponding author.
